# Glycemic control and the risk of tuberculosis in patients with diabetes: A cohort study in a Mediterranean city

**DOI:** 10.3389/fpubh.2022.1017024

**Published:** 2022-11-17

**Authors:** Violeta Antonio-Arques, Joan A. Caylà, Jordi Real, Antonio Moreno-Martinez, Àngels Orcau, Didac Mauricio, Manel Mata-Cases, Josep Julve, Elena Navas Mendez, Rai Puig Treserra, Joan Pau Millet, Jose Luis Del Val García, Bogdan Vlacho, Josep Franch-Nadal

**Affiliations:** ^1^DAP-Cat Group, Unitat de Suport a la Recerca Barcelona, Fundació Institut Universitari per a la Recerca a l'Atenció Primària de Salut Jordi Gol i Gurina (IDIAPJGol), Barcelona, Spain; ^2^Primary Health Care Center La Ràpita - Alcanar, Gerència d'Atenció Primària Terres de l'Ebre, Institut Català de la Salut, Tortosa, Spain; ^3^Tuberculosis Research Unit Foundation of Barcelona, Barcelona, Spain; ^4^CIBER of Diabetes and Associated Metabolic Diseases (CIBERDEM), Instituto de Salud Carlos III, Madrid, Spain; ^5^Department of Infectious Diseases, Hospital Clínic de Barcelona, Barcelona, Spain; ^6^CIBER of Epidemiology and Public Health (CIBERESP), Instituto de Salud Carlos III (ISCIII), Madrid, Spain; ^7^Epidemiology Service, Agència de Salut Pública de Barcelona, Barcelona, Spain; ^8^Department of Endocrinology and Nutrition, Hospital Universitari de la Santa Creu i Sant Pau, Barcelona, Spain; ^9^Department of Medicine, University of Vic—Central University of Catalonia, Barcelona, Spain; ^10^Primary Health Care Center La Mina, Gerència d'Atenció Primària Barcelona Ciutat, Institut Català de la Salut, Barcelona, Spain; ^11^Department of Biochemistry, Institut de Recerca de l'Hospital de la Santa Creu i Sant Pau, Barcelona, Spain; ^12^Fundació Institut Universitari per a la Recerca a l'Atenció Primària de Salut Jordi Gol i Gurina (IDIAPJGol), Barcelona, Spain; ^13^Unitat d'Avaluació, Sistemes d'informació i Qualitat, Gerència d'Àmbit d'Atenció Primària Barcelona Ciutat, Institut Català de la Salut, Barcelona, Spain; ^14^Primary Health Care Center Raval Sud, Gerència d'Atenció Primària Barcelona Ciutat, Institut Català de la Salut, Barcelona, Spain

**Keywords:** comorbidities, diabetes mellitus, diabetes complications, glycemic control, Primary Health Care, social determinants of health, tuberculosis

## Abstract

**Background:**

Diabetes mellitus (DM) is one of the leading chronic diseases globally and one of the most common causes of death, morbidity, and poor quality of life. According to the WHO, DM is also one of the main risk factors for developing active tuberculosis (TB). Subjects with DM are at a higher risk of infections, in addition to frequent micro and macrovascular complications, and therefore sought to determine whether poor glycemic control is linked to a higher risk of developing TB.

**Methods:**

We used a retrospective cohort of diabetic subjects to predict the incidence of TB. All DM patients were recruited from Ciutat Vella (the inner-city of Barcelona) from January 2007 until December 2016, with a follow-up period until December 2018 (≥2 years). Data were extracted from Barcelona's Primary Care medical record database - SIDIAP, and linked to the Barcelona TB Control Program. The incidence of TB and the impact of glycemic control were estimated using time-to-event curves analyzed by Cox proportional hazard regression. Hazard ratios (HRs) and 95% confidence intervals (CIs), unadjusted and adjusted by potential confounding variables, were also assessed, which included age, sex, diabetes duration, macrovascular and microvascular signs, BMI, smoking habit, alcohol consumption and geographical origin.

**Results:**

Of 8,004 DM patients considered for the study (equating to 68,605 person-years of follow-up), 84 developed TB [incidence rate = 70 (95% CI: 52–93) per 100,000 person-years]. DM subjects with TB were younger (mean: 52.2 vs. 57.7 years old), had higher values of glycosylated hemoglobin (HbA1c) (7.66 vs. 7.41%) and total triglycerides (122 vs. 105 mg/dl), and had twice the frequency of diabetic nephropathy (2.08 vs. 1.18%). The calculated incidence rate increased with increasing HbA1c: 120.5 (95% CI 77.2–179.3) for HbA1c ≥ 7.5%, 143 (95% CI 88.3–218.1) for HbA1c ≥ 8% and 183.8 (95% CI 105–298) for HbA1c ≥ 9%. An increase in the risk of TB was also observed according to a poorer optimization of glycemic control: adjusted HR 1.80 (95% CI 0.60–5.42), 2.06 (95% CI 0.67–6.32), and 2.82 (95% CI 0.88–9.06), respectively.

**Conclusion:**

Diabetic subjects with worse glycemic control show a trend toward a higher risk of developing TB.

## Introduction

Diabetes mellitus (DM) is one of the leading chronic diseases in the world (1) and one of the most common causes of death (2). Diabetes-associated mortality particularly impacts patients in low and middle-income countries due to poor control of the disease. Type 2 DM (T2DM) is the most frequent type of DM, accounting for ~90% of DM worldwide (3).

In patients with DM, chronic hyperglycemia underlies the development of macrovascular and microvascular complications, these complications are associated with the greatest burden on patients, caregivers and the health system (4–6). The aim of DM management is to prevent the development of these complications by achieving and maintaining glycemic control. A legacy effect is already known, i.e., early intensive optimization of glycemic control is frequently associated with a favorable reduction in the risk of myocardial infarction, death from any cause and microvascular disease, with the benefits of improved glycemic control in newly diagnosed patients maintained over time (7).

Besides vascular complications, DM has also been linked to an increased incidence of infections (8, 9), especially those related to lower respiratory, gastrointestinal, and urinary tract infections, and bacterial and mycotic skin and mucous membrane infections (8, 10). Current evidence suggests that DM patients with a poorer glycemic control are at an increased risk of these infections and consequent hospitalization and mortality (11, 12). In addition to the infections mentioned above, DM is considered an important risk factor for developing tuberculosis (TB) (2, 13), and poor glycemic control has been associated with a higher risk of developing this infection and worse outcomes (14–16). Moreover, patients with DM have an increased risk of cavitary pulmonary TB and hospitalization (17) and more risk of treatment failure, death, relapse and multidrug resistant TB (MDR-TB) (18).

Both DM and TB can be considered syndemic conditions (19). Countries with an increasing prevalence of DM also have a higher incidence of TB, especially seen in South-East Asia (2). Therefore, this clinical issue has become a priority for the World Health Organization (WHO) in recent years (20). Focusing on Europe, although most countries have a low TB incidence, inner-city districts of big cities, usually disadvantaged urban areas, show a higher notification rate (21).

In the current study, we used data from the diabetic population of Ciutat Vella, an inner-city district of Barcelona that has a three times higher incidence of TB than other city districts (22).

We aimed to estimate the incidence and characteristics of TB in patients with DM according to different HbA1c thresholds to analyze if higher values of HbA1c are linked to a higher risk of developing TB.

## Materials and methods

### Study design

We performed a subanalysis from a retrospective observational cohort. The methodology related to the primary data has been previously published (23). In the present study, we selected a cohort of diabetes patients registered between January 1st, 2007 and December 31st, 2016, with a follow-up period until December 2018. The inclusion date was January 1st, 2007, but for those participants who became diabetic during the study period the date of diagnosis was used.

### Study population and sample

**Figure 1:**
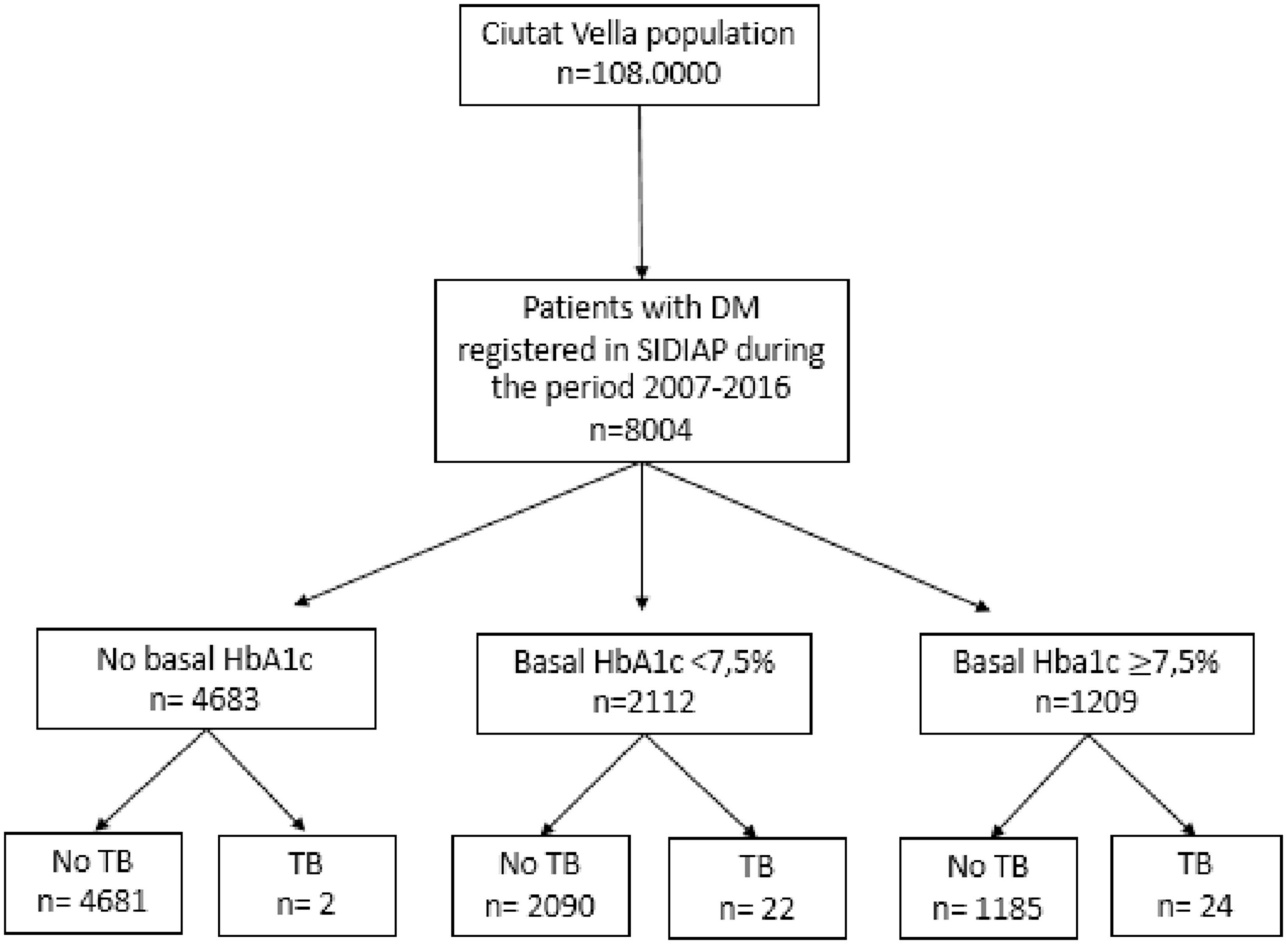
Flow chart of the DM cohort within the study population.

The primary study was carried out in the district of Ciutat Vella, the inner city of Barcelona, with a population of 108,000, that is characterized by a lower socioeconomic level and a higher percentage of immigrants (50.1%), compared to the rest of the city (24), and also has the highest incidence of infectious diseases (25). A cohort of 8,004 subjects with diabetes was analyzed in this study (Figure 1).

### Inclusion and exclusion criteria

Patients over 18 years who fulfilled the criteria for a DM diagnosis were included. People younger than 18 years or without appointments registered in their Primary Health Care Centre during the study period were excluded.

### Data sources

All data were treated as confidential according to the ethical principles of the Helsinki Declaration of 1964, revised by the World Medical Organization in Fortaleza, Brazil, in 2013, and the Spanish Organic Law 3/2018 of Data Protection. Clinical characteristics and specific data on DM were extracted from the Primary Care medical record database from Barcelona. Specific data on TB were obtained from the Barcelona TB Prevention and Control Program. Both sources were linked through an anonymized unique identifier. Patients' informed consent was unnecessary because the databases consisted of pseudo-anonymized data. This study obtained an Ethics Committee approval in 2016 (code P16/023).

### Variables

The primary outcome was defined as the occurrence and date of a TB diagnosis during the follow-up period.

We obtained the following information on the subjects at baseline: (1) demographic characteristics, smoking habits, and excessive alcohol consumption [defined by the recorded International Classification of Disease 10 (ICE.10) F10.1], (2) clinical variables, (3) laboratory data, (4) history of comorbidities.

When analyzing the country of origin, those patients from South-East Asia (Pakistan, Bangladesh, and India) (24) were grouped in one category: Hindustani origin.

We considered subjects to have DM if they had an ICD-10 (International Classification of Diseases) diagnostic code for DM in their medical record (E10–E14) or if they were taking any class of antidiabetic drug other than metformin (sometimes used to treat other conditions). Diabetes-related information was collected including glycated hemoglobin (HbA1c) during the follow-up, DM duration and treatment (i.e., lifestyle management, oral medication: metformin, secretagogues, DPP4-I, ISGLT2, arGLP1, and/or insulin), microvascular (neuropathy, nephropathy, retinopathy) and macrovascular complications (stroke, peripheral arteriopathy, and ischemic heart disease), and heart failure. As per the Barcelona TB Program, an active case of TB was considered when the study subject had a maintained prescription of an anti-TB drug. The diagnosis of TB was based on the recommendations of the Spanish Consensus (26). The priority method was the bacteriological study (baciloscopy and/or culture), but when this was not possible, PCR techniques, histology, biochemical methods (ADA) were used, or radiological and/or clinical impression.

This program has an active epidemiological surveillance system that makes under-detection very unlikely, with a good follow-up of patients that includes the recording of TB cases and their characteristics, diagnostic procedure (smear observation, culture, tuberculin skin test), and location (pulmonary or extrapulmonary) and treatment. The socioeconomic deprivation index (MEDEA) was collected in patients with TB. This index classifies each study subject by living area, whereby values with an average of 0 and a standard deviation of 1 and higher values indicate a more unfavorable socioeconomic situation (27).

### Statistical methods

Baseline comorbidities and demographic and clinical characteristics were described by frequencies (*n*) and percentages (%). Continuous variables were described using mean and standard deviation (SD). Comparison tests were computed as appropriate (*t*-test, and Fisher's exact test) using compareGroups R package (version 4.5.1) (28). In Kaplan-Meier analysis time-varying HbA1c was considered.

We conducted a time-to-event analysis to estimate the incidence of TB globally and by HbA1c group. Time-dependent variables with Cox models were performed to examine the association between glycemic control levels and the risk of TB (unadjusted and adjusted for confounding variables, as appropriate). A different grouping strategy was assessed, and risk profiles were constructed based on HbA1c continuous and risk cut-off points: ≥7.5%, ≥8%and ≥9%, respectively, according to local guidelines (29).

A database was constructed for the time-dependent models, and HbA1c values were updated every time patients had a new value available. Thus, the last HbA1c was carried over to the next value (or change) or the end of the follow-up (TB event, death, or end of study).

Cox proportional risk regression models for clustered data with constant and varying (HbA1c) variables over time were fitted using the *survival* R package (30). Hazard ratios (HR), unadjusted and adjusted, with their 95% confidence intervals (CIs), were estimated. CIs and *p*-values were computed with robust standard errors to account for cluster (subjects).

A sensitivity analysis of different fitted models, including different adjusted variables, was performed. A complete-cases analysis was performed with values available. Statistical significance was established as a *p*-value < 0.05. Data management and analysis were performed with the R version 3.6.3 package (31).

## Results

### General characteristics

A total of 8,004 diabetes subjects were analyzed, equating to 68,605 person-years of follow-up (PYFU), with 48 developing TB during the follow-up period. Compared to DM subjects without TB, DM subjects with TB were younger (52.2 vs. 57.7 years old), had higher levels of triglycerides (122 vs. 105 mg/dl), had a higher prevalence of diabetic nephropathy and neuropathy, and were more frequently from Hindustan (29.2 vs. 13.4%) (Table 1).

**Table 1:** Baseline characteristics of the study variables in diabetic patients without and with the occurrence of tuberculosis during the follow-up period.

**Variable**	**Type 2 DM without TB** **(*n* = 7,956)**	**Type 2 DM with TB** **(*n* = 48)**	***p*** **-values**
**Age** (years) mean (SD)	57.7 (14.2)	52.2 (13.4)	0.007^a^
**Gender**: *n* (%)			0.353^b^
Men	4,865 (61.1%)	33 (68.8%)	
Women	3,091 (38.9%)	15 (31.2%)	
**Origin**			0.008^b^
Spain/high-income countries	3,172 (39.8%)	17 (35.4%)	
Hindustan	1,069 (13.4%)	14 (29.2%)	
Other	965 (12.1%)	7 (14.6%)	
Unknown	2,750 (34.6%)	10 (20.8%)	
**Alcohol abuse**	398 (5.0%)	3 (6.25%)	0.733^b^
**Smoking habit**	587 (7.38%)	1 (2.08%)	0.260^b^
**Homeless**	6 (5.94%)	3 (6.25)	0.837^b^
**Unemployment**	9 (14.5%)	15 (32.6%)	<0.001^b^
**High social risk** (Medea > 2.3)	50 (42.0%)	12 (34.3%)	0.541^b^
**Duration of DM** (years) mean (SD)	3.22 (5.77)	2.59 (4.04)	0.280^a^
**Total medical appointments**	86.2 (80.7)	115 (84.1)	0.021^a^
**HbA1c** (%) mean (SD)	7.41 (1.85)	7.66 (2.24)	0.613^a^
HbA1c ≥ 7.5%	1,200 (36.3%)	11 (55%)	0.135^b^
HbA1c ≥ 8%	906 (27.4%)	6 (30%)	0.996^b^
HbA1c ≥ 9%	565 (17.1%)	5 (25%)	0.271^b^
**BMI** (kg/m^2^) mean (SD)	30.1 (5.30)	29.8 (4.54)	0.798^a^
BMI < 25	427 (15.9%)	0	0.077^b^
BMI 25–30	1,010 (37.6%)	9 (64.3%)	
BMI > 30	1,249 (46.5%)	5 (35.7%)	
**SBP** (mmHg) mean (SD)	137 (17.8)	135 (19.9)	0.570^a^
**DBP** (mmHg) mean (SD)	79.3 (11.2)	78.5 (12.5)	0.791^a^
**Total cholesterol** (mg/dl) mean (SD)	207 (48.0)	205 (45.7)	0.850^a^
**HDL cholesterol** (mg/dl) mean (SD)	48.1 (13.5)	45.5 (7.62)	0.165^a^
**LDL cholesterol** (mg/dl) mean (SD)	124 (36.2)	131 (42.4)	0.504^a^
**Triglycerides** (mg/dl) mean (SD)	105 (27.2)	122 (21.2)	0.025^a^
**Hemoglobin**	13.5 (1.24)	12.7 (1.57)	0.101^a^
**Platelets**	259 (71.4)	326 (80.4)	0.138^a^
**ESR**	22.9 (19.1)	40.6 (43.1)	0.411^a^
**DM complications**			
Macrovascular disease	640 (8.04%)	4 (8.33%)	0.793^b^
Diabetic retinopathy	296 (3.72%)	2 (4.17%)	0.699^b^
Diabetic nephropathy	94 (1.18%)	1 (2.08%)	0437^b^
Diabetic neuropathy	95 (1.19%)	1 (2.08%)	0.115^b^
Heart failure	221 (2.78%)	1 (2.08%)	1.000^b^
**DM treatment**			
Non-pharmacological	5,197 (65.3)	31 (64.6)	0.909^b^
Metformin	2,293 (28.8%)	15 (31.2%)	0,833^b^
Secretagogues	826 (10.4%)	6 (12.5%)	0.632^b^
DPP4-i	7 (0.1)	0	0.837^b^
iSGLT2	1 (0)	0	0.938^b^
Insulin	550 (6.91%)	3 (6.25%)	0.854^b^

BMI, body mass index; DBP, diastolic blood pressure; DPP4-i, inhibitors of dipeptidyl peptidase 4; ESR, erythrocyte sedimentation rate; HDL, high-density lipoprotein; iSGLT2, Sodium-glucose cotransporter type 2 inhibitors; LDL, low-density lipoprotein; SBP, systolic blood pressure; SD, standard deviation.

a *t*-test.

b Fisher's exact test.

### Differences on TB characteristics

Overall, baseline HbA1c values were available for 46 DM subjects with TB. No statistically significant differences were observed in subjects with an HbA1c ≥ 7.5% vs. those with HbA1c values < 7.5% for localization of TB disease, radiography and tuberculin skin test (Table 2).

**Table 2:** Characteristics of tuberculosis patients according to basal glycemic control.

**Variable**	**HbA1c < 7.5%** **(*n* = 22)**	**HbA1c ≥7.5%** **(*n* = 24)**	***p*** **-values^a^**
**Localization**			1.000
Pulmonary	16 (72.7%)	17 (70.8%)	
Extrapulmonary	6 (27.3%)	7 (29.2%)	
**Radiography**			0.575
Normal	6 (27.3%)	7 (29.2%)	
Cavitary	2 (9.09%)	5 (20.8%)	
Anormal non-cavitary	13 (59.1%)	12 (50%)	
**TST**			0.683
Positive	7 (31.8%)	9 (37.5%)	
Negative	2 (9.09%)	3 (12.5%)	
Unknown	13 (59.1%)	12 (50%)	
**Bacteriology**			0.683
Positive culture	11 (50%)	8 (33.3%)	
Negative culture	3 (13.6%)	7 (29.2%)	
ADA	1 (4.55%)	1 (4.17%)	
PCR (+)	0 (0%)	1 (4.17%)	
**DM treatment**			1.000
Non-pharmacological treatment	13 (59.1%)	14 (58.3%)	
NIAD	8 (36.4%)	9 (37.5%)	
Insulin	1 (4.55%)	1 (4.17%)	

a Fisher's exact test.

TST, tuberculine skin test; NIAD, Non-insulin antidiabetic treatment; ADA, adenosine deaminase; PCR, protein chain reaction.

At the time of TB, 88.8% were symptomatic, and 44% were smear-positive (21.3% were smear-negative, but the culture was positive).

Regarding treatment, the most commonly used regimen was the standard treatment with 4 drugs (47.2%), followed by 6 months with 3 drugs (11.2%). In 13.5% of the cases had resistant TB.

### Risk of developing TB according to glycemic control

**Figure 2:**
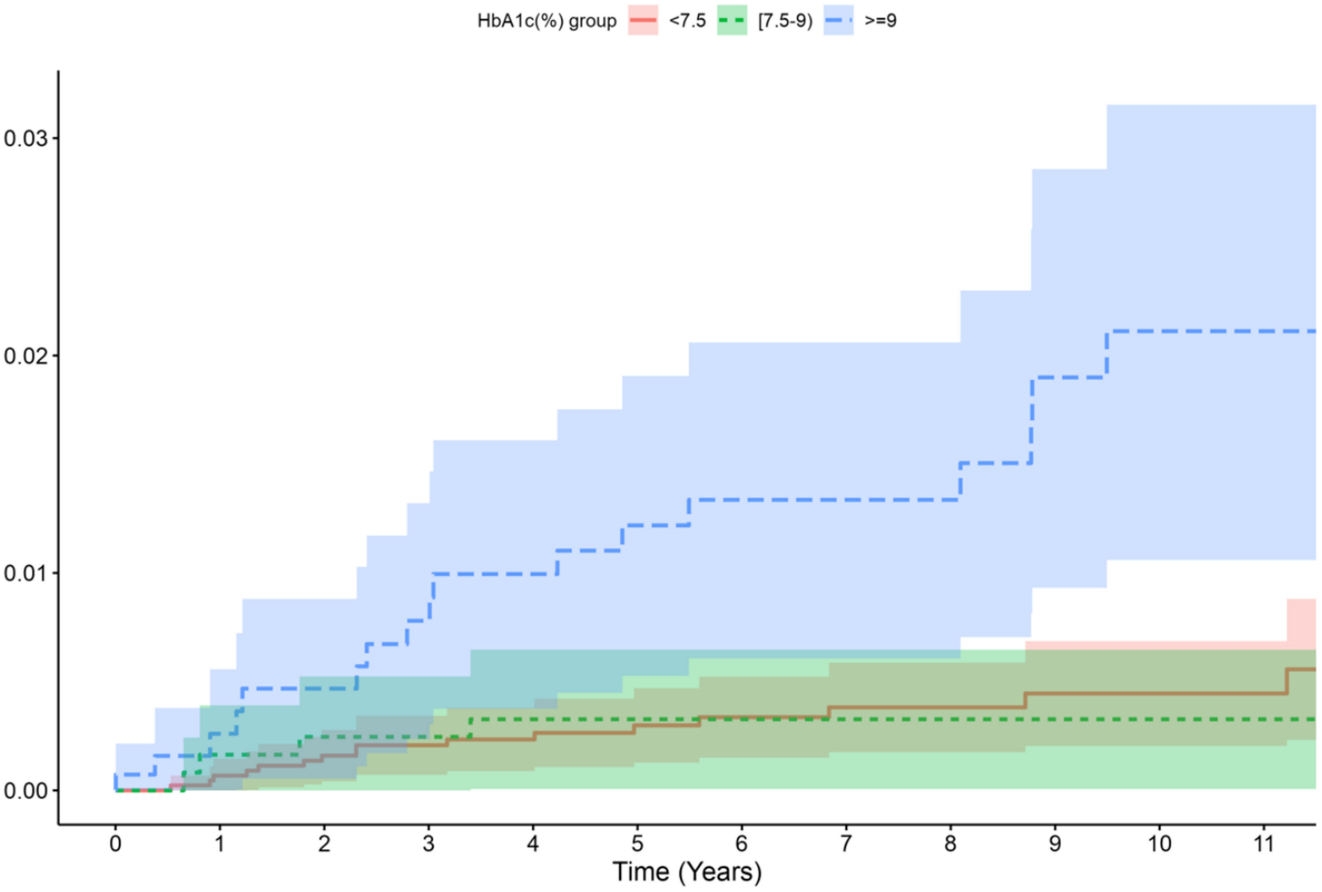
Cumulative incidence per 100,000 person-year of TB according to HbA1c level.

The TB incidence rate was 70 per 100,000 PYFU in the overall DM group, and was 90 per 100,000 in the DM group with any HbA1c available. The incidence of TB increased with increasing HbA1c cut-off values (i.e., highest in the group with HbA1c ≥9% followed by ≥8% and then ≥7.5%) (Table 3). The incidence curves for TB over time according to the HbA1c group also showed that the group with HbA1c ≥ 9% was more prone to TB than the other HbA1c groups (Figure 2).

**Table 3:** Incidence and risk of TB (unadjusted and adjusted hazard ratio) in DM patients depending on HbA1c level (%).

	**Total person** **time (years)**	**Number** **of TB** **cases**	**Incidence** **rate per** **100,000 p –y**	**95% CI**	**HR unadjusted**		**HR adjusted** **model 1**		**HR adjusted** **model 2**	
					**HR** **(95% CI)**	***p*** **-value**	**HR** **(95% CI)**	***p*** **-value**	**HR** **(95% CI)**	***p*** **-value**
All DM patients (*n* = 8,004)	68,605	48	69.97	51.6–92.8	–		–		–	
DM patients with HbA1c available (*n* = 6,733)	51,188	46	89.9	65.8–120.0	–		–		–	
HbA1c < 7,5%	31,276	22	70.3	44.1–106.5	Ref		Ref		Ref	
HbA1c ≥ 7,5%	19,912	24	120.5	77.2–179.3	**2.22** (1.16–4.28)	0.016	**1.87** (0.98–3.64)	0.064	**1.89** (0.60–5.42)	0.293
HbA1c < 8%	36,468	25	68.6	44.4–101.2	Ref		Ref		Ref	
HbA1c ≥ 8%	14,721	21	143.0	88.3–218.1	**2.50** (1.31–4.78)	0.005	**2.07** (1.07–4.01)	0.031	**2.06** (0.67–6.32)	0.207
HbA1c < 9%	42,481	30	70.6	47.6–100.8	Ref		Ref		Ref	
HbA1c ≥ 9%	8,707	16	183.8	105–298.4	**4.36 (**2.28–8.33)	<0.001	**3.62** (1.86–7.04)	<0.001	**2.82** (0.88–9.06)	0.082

**Model 1**: adjusted by age and sex.

**Model 2**: adjusted by age, sex, years of evolution of DM, microvascular and macrovascular complications, alcohol, smoking habit and geographical origin. The bold values are the hazard ratios for each HbA1c thresholds in each model.

**Figure 3:**
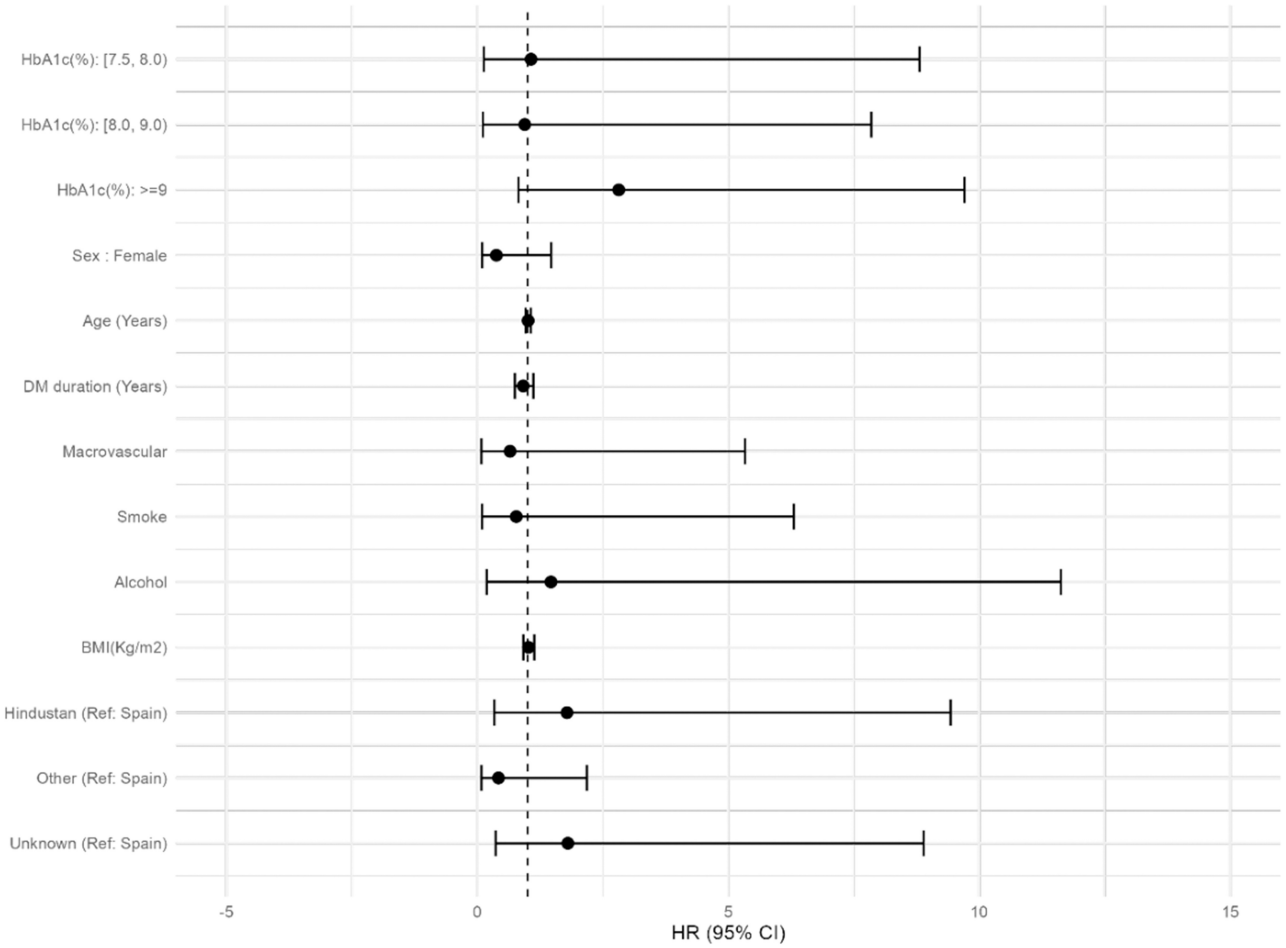
Forest plot of the factors associated with DM that can influence the appearance of TB.

The association between HbA1c and risk of TB according to the different HbA1c cut-off points (HbA1c ≥ 7.5, 8, and 9%) was further analyzed by estimating the unadjusted and adjusted HR: with higher levels of glycated hemoglobin, a higher incidence of TB was observed, reaching the highest values in those subjects with values over 9%. When we adjusted by age and sex (model 1), we found statistically significant differences if HbA1c ≥ 8 and ≥9%, but not when adjusting by age, sex, years of evolution of DM, macrovascular complications, alcohol or smocking habit, BMI and geographical origin (model 2). Overall the risk increased with higher values of HbA1c (Table 3; Figure 3).

## Discussion

DM is one of the main risk factors for developing TB (2), and both diseases frequently coexist, even in countries with a low incidence of TB (32). In our work, we observed an increased incidence of TB specially in patients with DM with higher values of HbA1c; the increased HRs also suggested an increased risk. This study took place in an inner city district of Barcelona, where we found in previous work that DM patients had 1.90 (CI: 1.18-3.07) higher risk of developing TB (23). Without considering the metabolic control, a prospective cohort study showed that DM patients had more than 2-fold risk of developing TB, and this risk increased with increasing number of DM complications (33).

The higher risk of TB in patients with DM and poor metabolic control has been studied before (34–36). In a cohort study conducted in Taiwan with 120,000 patients, with a multivariate analysis, it was observed that diabetic patients with poor glycemic control [described as a basal fasting plasma glucose (FPG) > 130 mg/dl] had twice the risk of developing TB than non-diabetic patients. While there was no difference in the risk of TB between non-diabetic subjects and diabetic patients with good glycemic control, the risk of TB increased with increasing FPG in diabetic patients (14). The results also align with another study conducted in China at the hospital level, where patients with poor metabolic control (also defined as basal FPG > 130 mg/dl) were 2.66 times more likely to develop pulmonary TB (37), and with another work also performed in China, where patients with DM showed higher risks of active (3.11), culture-confirmed (3.08), and pulmonary (3.63) but not extrapulmonary tuberculosis with baseline hemoglobin A1c > 7% (38).

In addition, as well as influencing the onset of active TB, a poor glycemic control may also increase the risk of latent TB infection. According to this previous study, glycemic control assessed by glycosylated hemoglobin values influences the capacity of the host to control the infection by altering immune response, and had increased susceptibility to pathogens (39). Supporting this, in a recent meta-analysis, it was concluded that poor glycemic control (defined by an HbA1c > 7%) would double the prevalence of TB (40).

Other studies were conducted without finding any relation between the risk of TB and the metabolic control. In a population-based study placed in Denmark, the risk of TB in patients with DM was lower than previously expected (HR 1.18) and no significant association with glycemic control was found. Probably the main limitation of this work was the low number of DM patients analyzed (7 subjects with HbA1c between 7 and 7.9%, 16 for HbA1c ≥ 8% and 20 subjects with unknown values of HbA1c) and the low probability of TB in low-incidence countries (41). With similar conclusions, a study performed in UK showed that the increased risk of TB among DM patients was 1.3, it was considered moderate, and no evidence for a higher risk linked to a worse glycemic control was observed, but DM patients with the lowest and highest rates of chronic disease management had a higher risk of TB (42).

The results of our work are in line with the publications mentioned above: although the results were not statistically significant, probably due to the small number of patients, the study was done in a high incidence of TB zone, and a relation can be observed: with worse glycemic control, the risk of TB is greater. In the inner cities of developed countries, always with a high incidence of TB, a good control of DM patients is necessary to avoid complications as TB.

When adjusting by age, sex, years of evolution of DM, microvascular and macrovascular complications, alcohol, smoking habit and geographical origin, the HR in the 9% cut-off point changes from 3.62 to 2.82. This fact can be explained because the addition of the variable “geographical origin”. People from Hindustan are the group with the highest incidence of TB in our city. They have much more latent TB [as they come from high incidence countries (2)] and, therefore, a greater risk of developing active TB.

Disruptions of immunological mechanisms because of hyperglycemia may explain the increased TB risk. In this respect, excess glucose may affect cell activation, phagocytic capacity, microbicide mechanisms of alveolar macrophages and neutrophils, leukocyte transmigration, and chemotaxis, potentially delaying antigen presentation and, consequently, activation of the necessary immunological mechanisms to fight against *Mycobacterium tuberculosis* (43). Of note, DM patients show the most severe clinical presentation of TB (17), and a higher risk of mortality and multi-drug resistant TB (18, 44).

Although we did not find significant differences between groups when when comparing pulmonary or extrapumonary TB and, on the other hand, the localization of the lesions detected *via* chest radiography, other studies have described differences in those with poorer glycemic control. In a study of more than 600 diabetic patients diagnosed with TB, patients with poor metabolic control, defined by HbA1c > 7%, had more severe TB, more extensive lung disease, and more cavitations (15). Another work also described more involvement of lower lung fields and increased likelihood of cavitation with poor glycemic control (45). In the work of Huang et al., diabetic patients with a Hb1Ac > 8% had more extensive lung lesions, atypical findings, more lymphadenopathy, more cavitation, and a greater likelihood of involvement of all lung lobes (16). Regarding symptoms, one study concluded that diabetic patients, especially those with HbA1c > 9%, had more clinical signs of TB: hemoptysis, asthenia, and weight loss (46). In our work, although differences were insignificant, patients with poor glycemic control had more cavitary lesions on chest-x-ray.

The main limitation of this work is the low number of TB cases found during the follow-up period but our inner-city has a high incidence of TB compared with the rest of the city (22) what facilitated this study. Fortunately, TB is not a frequent disease nowadays in Western European countries but its incidence in the inner cities of developed countries is still too high (47). Moreover, we limited the selection of subjects to those with both diseases: DM and TB. It is a very concrete group of subjects, but, in our opinion, this analysis has a clinical and epidemiological value. According to the literature, other studies which reported a relationship between glycemic control and TB had a moderate number of subjects with both TB+DM diseases: 63 and 214 in two studies in Taiwan (14, 16), and 105 in a study in China (37). Within the limitations of working with real world data, we could not have a systematic follow-up of glycated hemoglobin as this was a retrospective study. The small number of cases with TB has made a clear statistical significance difficult. Working in a real-world-data environment may have some inherent limitations, but it has the great strength of reflecting the conditions of standard clinical practice in almost all patients.

Studies with large databases may involve different types of bias. Our study has tried to minimize the diagnostic errors of DM and TB by basing their diagnosis both on the ICD-10 clinical coding and on the incorporation of complementary variables such as the use of specific treatment for these pathologies. This method has been previously validated and published with our database (48), but we assume that, in some cases, it may be a limitation. On the other hand, the fact that some data on the bacteriology of TB diagnosis is not available is a limitation, so the diagnosis has been complemented with other variables, as detailed in the Section Methods.

## Conclusion

In summary, poor glycemic control is related to an increased risk of TB development. Given the results of this research, improved glycemic control can be pursued in DM not only to reduce the risk of vascular events but also to decrease the risk of TB and its complications, especially in areas with a high prevalence of TB. The increasing worldwide prevalence of DM and failures to eradicate TB, coupled with the synergy between DM and TB, make it necessary to consider managing these two diseases together.

## Data availability statement

The datasets analyzed for this study can be found in the following link: https://github.com/USR-DAPCAT/TBC_Glicada.

## Ethics statement

The studies involving human participants were reviewed and approved by Primary Health Care University Research Institute Jordi Gol, in 2016 (Code: P16/023). Written informed consent for participation was not required for this study in accordance with the national legislation and the institutional requirements.

## Author contributions

VA-A, JC, JF-N, JR, ÀO, and AM-M participated in the study design. ÀO, JD, and JR worked on data collection. JR, EN, RP, and JF-N performed all statistical work. VA-A, JF-N, and JC were major contributors in writing the manuscript. MM-C, DM, JJ, JM, BV, and AM-M reviewed and corrected the manuscript. BV contributed to prepare the manuscript according to the journal policies. All authors contributed to the article and approved the submitted version.

## Funding

This work was supported by grants given by the Instituto de Salud Carlos III PI16/01751 (Spanish Ministry of Economy), the Institut Universitari per a la Recerca a l'Atenció Primària de Salut Jordi Gol i Gurina (Catalan Health Institute) PREDOC_ECO-19/2, and the Fundación redGDPS (Beca de apoyo José Luis Torres a la Investigación).

## Conflict of interest

The authors declare that the research was conducted in the absence of any commercial or financial relationships that could be construed as a potential conflict of interest.

## Publisher's note

All claims expressed in this article are solely those of the authors and do not necessarily represent those of their affiliated organizations, or those of the publisher, the editors and the reviewers. Any product that may be evaluated in this article, or claim that may be made by its manufacturer, is not guaranteed or endorsed by the publisher.
